# CSF/serum IL-6 ratio and its correlation with mri severity in mog antibody-associated cerebral cortical encephalitis: an exploratory pilot study

**DOI:** 10.3389/fimmu.2026.1921290

**Published:** 2026-08-31

**Authors:** Honglian Zhang, Xiaoqing Lu, Jie Li, Mengting Xiong, Dan He, Zicong Li, Wentao Dong, Xiaomu Wu, Gang Huang

**Affiliations:** 1 Jiangxi Provincial People’s Hospital, The First Affiliated Hospital of Nanchang Medical College, Nanchang, Jiangxi, China; 2 Xiangya Hospital, Central South University, Jiangxi, National Regional Center for Neurological Diseases, Nanchang, Jiangxi, China; 3 The First Affiliated Hospital of Nanchang University, Nanchang, Jiangxi, China

**Keywords:** CNS compartmentalized inflammation, CSF IL-6, CSF/serum IL-6 ratio, MOG-CCE, MRI score

## Abstract

**Objective:**

We aimed to quantify the relationship between CSF IL-6 compartmentalization and MRI lesion extent in patients with MOG-CCE, and to evaluate the CSF/serum IL-6 ratio as a biomarker of CNS-compartmentalized inflammation.

**Methods:**

Eight MOG-CCE patients (≥14 years old) were retrospectively enrolled. CSF and serum cytokines (IL-6, IL-17, IFN-γ) and MRI scans were collected. A semi-quantitative MRI scoring system (0–9) was developed; inter-rater reliability was assessed by two independent radiologists. Associations were evaluated using Spearman correlation and simple linear regression.

**Results:**

All patients presented with seizures at onset. Median CSF IL-6 (284.53 pg/mL) markedly exceeded paired serum levels (2.25 pg/mL; Z = 2.521, p = 0.012; Cohen’s d = 1.084). The median CSF/serum IL-6 ratio was 220.33 (IQR: 64.26–690.89), exceeding 1 in all patients. The MRI scoring system showed good inter-rater reliability (87.5% concordance; P = 0.893). The total MRI score correlated significantly with CSF IL-6 (r = 0.819, p = 0.013) and the CSF/serum IL-6 ratio (r = 0.807, p = 0.015). Simple linear regression confirmed that the Ln-transformed ratio predicted MRI score (R² = 0.573, p = 0.030, B = 0.659, β = 0.757). Crucially, the ratio showed no significant correlation with conventional systemic inflammatory markers.

**Conclusion:**

CSF IL-6 levels correlated significantly with MRI severity in MOG-CCE. The Ln-transformed CSF/serum IL-6 ratio explained approximately 57.3% of MRI variance and was uncorrelated with systemic inflammatory indices, supporting its specificity for CNS-compartmentalized immune activation. These findings position the CSF/serum IL-6 ratio as a candidate quantitative biomarker of local CNS inflammatory intensity and lesion burden in MOG-CCE. As an exploratory, hypothesis-generating pilot study (n = 8), these results require prospective validation in larger cohorts with Reiber graph analysis before the ratio can be adopted as a clinical biomarker.

## 1 Introduction

Myelin oligodendrocyte glycoprotein antibody-associated disease (MOGAD) is an immune-mediated demyelinating disorder of the central nervous system. Its phenotypic spectrum has gradually emerged from the initial taxonomic confusion with neuromyelitis optica spectrum disorder (NMOSD) and is now recognized as a distinct autoimmune disease entity encompassing a broad range of clinical presentations. The 2023 international diagnostic consensus (1) formally established cerebral cortical encephalitis (CCE) as a core MOGAD phenotype, thereby marking the first systematic characterization of this subtype. Clinico-radiographically, adult MOG antibody-associated cerebral cortical encephalitis (MOG-CCE) is defined by epileptic seizure onset and cortical FLAIR hyperintensity with leptomeningeal enhancement—a correspondence that has been consistently replicated across multiple case series (2–4). Yet, beneath this phenotypic consensus, a fundamental question remains unresolved: the existing literature has largely confined cytokine analysis to qualitative statements on interleukin-6 (IL-6) involvement in immune mechanisms (5, 6), leaving a critical quantitative evidence gap between the intensity of inflammatory biology and the extent of radiographic injury.

A deeper examination reveals that the role of IL-6 in MOGAD inflammatory pathogenesis rests on two intertwined but insufficiently reconciled lines of evidence. The first line emerges from autoimmune encephalitis research. Levraut et al. (7) systematically demonstrated intrathecal elevation of cerebrospinal fluid (CSF) IL-6, suggesting that local CNS IL-6 production may serve as a sensitive indicator of compartmentalized immune activation. However, definitive confirmation of intrathecal synthesis requires Reiber graph analysis to exclude the confounding contribution of blood–brain barrier disruption (8). The second line concerns humoral and cytokine mechanisms of MOGAD pathophysiology. Misu et al. (5) anchor the pathology in antibody-mediated myelin damage, while Grebenciucova et al. (6) argue that, acting through the cytokine–demyelination cascade, IL-6 released by CNS astrocytes and microglia drives downstream demyelination and leads to oligodendrocyte and axonal injury. The coexistence of these two perspectives exposes a central tension: is IL-6 in MOGAD merely an epiphenomenon of inflammation, or is it an effector molecule that actively drives tissue injury? The answer to this question will directly determine whether CSF IL-6 can be established as a quantitative biomarker of lesional expansion.

To address this question, and drawing on the established biological role of IL-6 in compartmentalized CNS immune activation (7), we hypothesized that the degree of intrathecal IL-6 enrichment could serve as a quantitative biomarker of both inflammatory intensity and magnetic resonance imaging (MRI)-lesion burden in MOG-CCE. This exploratory, hypothesis-generating pilot study aimed to integrate fragmentary clinical observations into a coherent evidence chain and to provide a quantitative foundation for individualized management of MOG-CCE.

## 2 Subjects and methods

### 2.1 Study design

This single-center, retrospective observational study was conducted between January 2021 and July 2025. The study protocol was approved by the Ethics Review Committee of Jiangxi Provincial People’s Hospital. Given its retrospective nature, individual informed consent was waived; all clinical data were obtained and handled in accordance with the hospital’s confidentiality policies and the Declaration of Helsinki, and were anonymized prior to analysis.

### 2.2 Study subjects

We retrospectively reviewed the clinical records of MOGAD patients admitted to the Department of Neurology, Jiangxi Provincial People’s Hospital between January 2021 and July 2025. Inclusion criteria were: (1) meeting the 2023 International MOGAD Expert Panel diagnostic criteria (1), with the phenotype confined to CCE; (2) positive for anti-MOG antibodies in both serum and CSF by cell-based assay, with paired CSF and serum samples available; (3) acute-phase brain MRI demonstrating cortical T2-FLAIR hyperintense lesions; (4) age at onset ≥14 years; and (5) complete clinical data with at least six months of follow-up. Exclusion criteria were: (1) an alternative definite etiology explaining the clinical presentation; (2) coexisting positivity for other neurological autoantibodies; (3) concurrent MOGAD phenotypes with overlapping symptomatology; and (4) missing clinical or imaging data, or loss to follow-up.

### 2.3 Data collection

Standardized data extraction was performed via the hospital’s electronic medical record system. The following data were retrieved: (1) demographic and clinical characteristics—age, sex, presenting symptoms, and neurological signs; (2) laboratory findings—hematological parameters, CSF routine analysis, CSF biochemistry, CSF opening pressure, OCB, MOG-IgG titers (cell-based assay), and inflammatory cytokine levels (IL-6, IL-17, IFN-γ), the latter measured using the Qingdao Recovery–Mindray BriCyte E6 flow cytometer with a 12-plex cytokine detection kit; CSF and serum samples were collected in parallel during the acute phase and tested synchronously; and (3) neuroimaging—3.0 T MRI sequences including T1-weighted, T2-weighted, FLAIR, and contrast-enhanced imaging.

### 2.4 MRI scoring system

To quantify the extent of CNS involvement, we devised a cortical involvement scoring system (0–9) comprising the following components: (1) extent of cortical involvement (0–4)—ranging from no cortical lesion to bilateral extensive involvement; (2) deep structure involvement (0–2)—involvement of white matter, deep gray matter, or brainstem; and (3) enhancement characteristics (0–3)—degree of leptomeningeal enhancement. Detailed grading criteria are presented in **Table 1**.

**Table 1:** MRI scoring system for MOG-CCE.

Domain	Score	Description
Cortical Involvement	0–4	
	0	No cortical lesions
	1	Unilateral, focal cortical lesion (single gyrus)
	2	Unilateral, multi-lobar cortical lesions; OR bilateral, focal
	3	Bilateral, multi-lobar cortical lesions
	4	Bilateral, extensive cortical involvement (≥ 3 lobes per hemisphere)
Deep Structure Involvement	0–2	
	0	No deep structure involvement
	1	Involvement of subcortical or deep white matter (periventricular or centrum semiovale)
	2	Involvement of deep gray matter (thalamus, basal ganglia) or brainstem / cerebellum
Enhancement	0–3	
	0	No enhancement
	1	Mild, focal leptomeningeal enhancement
	2	Moderate, multi-lobar leptomeningeal enhancement
	3	Severe, extensive leptomeningeal enhancement

Total Score Range: 0–9 (sum of all three domain scores).

The scoring system was developed by the study group through consensus of two board-certified neuroradiologists with ≥5 years of experience, informed by prior radiological descriptions of MOGAD cortical/meningeal involvement and by analogous semi-quantitative imaging scales used in CNS demyelinating disease (9). The three domains (cortical, meningeal, and deep structure involvement) were selected to capture the clinico-radiographic features characteristic of MOG-CCE. The score has not yet been externally validated in an independent cohort.

Inter-rater reliability was assessed as follows. Two radiologists with at least five years of neuroimaging experience independently scored all scans under blinded conditions (masked to clinical information and each other’s scores). Agreement was evaluated by: (1) simple concordance rate—the proportion of cases with identical scores; and (2) the Wilcoxon signed-rank test, which compared the distribution of scores between the two raters (P < 0.05 considered statistically significant). Given the small sample (n = 8), a non-parametric approach was chosen (10). Score adjudication proceeded as follows: when scores differed by more than one point, a third senior radiologist determined the final score; when the difference was one point or less, the average of the two scores was taken.

#### 2.5 CSF/serum IL-6 ratio

The CSF/serum IL-6 ratio was defined as the quotient of CSF IL-6 concentration divided by serum IL-6 concentration, providing a preliminary index of IL-6 distribution within the CNS compartment. A ratio >1 indicated that CSF IL-6 exceeded the serum level. While Reiber graph analysis is the gold standard for assessing intrathecal synthesis (8), it could not be performed in the present study because serum albumin and QAlb were not measured; accordingly, the CSF/serum IL-6 ratio was adopted as a surrogate marker of intrathecal enrichment. CSF and serum IL-6 concentrations were quantified on a Qingdao Recovery–Mindray BriCyte E6 flow cytometer using a 12-plex cytokine detection kit (see Section 2.3), with paired samples collected and tested synchronously.

### 2.6 Treatment and follow-up

Acute-phase treatment regimens and maintenance immunotherapy strategies were documented. Follow-up was conducted via outpatient visits or telephone interviews to capture relapse events.

### 2.7 Statistical analysis

All statistical analyses were performed using SPSS 26.0. Continuous variables with a normal distribution (assessed by Shapiro-Wilk test) were reported as mean ± standard deviation; non-normally distributed variables were reported as median and interquartile range (IQR). For paired comparisons of CSF and serum cytokine levels, the Wilcoxon signed-rank test was applied; effect sizes were estimated using a Z-value–based Cohen’s d approximation (d = 2r/√(1 − r²), where r = Z/√n). Cohen’s d > 0.8 indicates large effect size; 0.5–0.8 indicates medium effect size; 0.2–0.5 indicates small effect size. Spearman rank correlation was employed to assess associations among the CSF/serum IL-6 ratio, MRI score, and other laboratory parameters. Because the IL-6 ratio followed a positively skewed distribution (Shapiro-Wilk P < 0.05) and regression residual diagnostics favored the Ln-transformed model, the ratio was natural-logarithm (Ln) transformed prior to linear regression. A simple linear regression model was then fitted to evaluate the predictive value of the Ln-transformed CSF/serum IL-6 ratio for the MRI score, with R², adjusted R², F-statistic, unstandardized coefficient (B), and standardized coefficient (β) reported. Given the exploratory nature of this study, no Bonferroni or false discovery rate (FDR) corrections were applied for multiple comparisons; accordingly, results from the correlation analyses should be interpreted as hypothesis-generating rather than confirmatory. A two-sided P < 0.05 was considered statistically significant.

## 3 Results

### 3.1 Demographic data and clinical characteristics

A total of 8 patients with MOG-CCE meeting the diagnostic criteria were enrolled. Mean age at onset was 28.50 ± 13.99 years; 6 patients (75.0%) were male and 2 (25.0%) were female. Seizure was the cardinal clinical feature—all 8 patients (100%) presented with seizures at disease onset. Other common manifestations included headache (5/8, 62.5%) and fever (3/8, 37.5%), and 6 patients (75.0%) exhibited positive meningeal signs. Notably, none of the patients developed psychiatric or behavioral abnormalities, motor deficits, visual impairment, or spinal cord involvement (**Table 2**).

**Table 2:** Demographic, clinical, and laboratory characteristics of patients with MOG-CCE.

Characteristics	Findings (N = 8)
Demographic Data	
Age at onset, years	28.50 ± 13.99
Sex, male, n (%)	6 (75.0)
Clinical Characteristics	
Seizure at onset, n (%)	8 (100)
Headache, n (%)	5 (62.5)
Fever, n (%)	3 (37.5)
Meningeal signs, n (%)	6 (75.0)
Routine hematological examinations	
Elevated WBC count, n (%)	5 (62.5)
WBC count, ×10^9^/L, mean ± SD	9.06 ± 1.92
Elevated NLR, n (%)	6 (75.0)
NLR, mean ± SD	4.05 ± 1.68
MLR, mean ± SD	0.30 ± 0.04
PLR, mean ± SD	165.97 ± 28.53
Routine CSF examinations	
CSF pleocytosis, n (%)	8 (100)
CSF WBC count, ×10^6^/L, median (IQR)	45.00 (27.50–160.00)
Elevated CSF protein, n (%)	4 (50.0)
CSF protein, mg/L, mean ± SD	623.12 ± 377.87
CSF pressure (cmH_2_O)	181.63 ± 54.87
Elevated CSF pressure, n (%)	4 (50.0)
Positive OCB, n (%)	4 (50.0)

### 3.2 Laboratory findings

#### 3.2.1 Routine hematological examinations

Routine hematological examinations revealed a mean leukocyte count of 9.06 ± 1.92 × 10^9^/L (reference: 3.5–9.5 × 10^9^/L), with 5 patients (62.5%) showing leukocytosis. The neutrophil-to-lymphocyte ratio (NLR) was 4.05 ± 1.68 (reference: 1–3), with elevation in 6 patients (75.0%); the monocyte-to-lymphocyte ratio (MLR) was 0.30 ± 0.04 (reference: 0.22–0.33); and the platelet-to-lymphocyte ratio (PLR) was 165.97 ± 28.53 (reference: 120–180) (**Table 2**).

#### 3.2.2 Routine CSF examinations

CSF analysis was performed in all 8 patients. All exhibited pleocytosis, with a median leukocyte count of 45 × 10^6^/L (IQR: 27.50–160.00 × 10^6^/L; reference: 0–10 × 10^6^/L). Elevated CSF protein was observed in 4 patients (50.0%), with a mean concentration of 623.12 ± 377.87 mg/L (reference: 150–450 mg/L). Mean CSF glucose was 3.03 ± 0.42 mmol/L (reference: 2.5–4.5 mmol/L), and mean CSF chloride was 125.38 ± 4.9 mmol/L (reference: 120.0–132.0 mmol/L). Metagenomic next-generation sequencing (mNGS) of CSF samples from all 8 patients detected no infectious pathogens. Mean CSF opening pressure was 181.63 ± 54.87 cmH_2_O (reference: 80–180 cmH_2_O), with 4 patients (50.0%) exhibiting elevated pressure. OCB testing, performed in 4 patients, was positive in all 4 tested cases (50.0% of the cohort) (**Table 2**).

#### 3.2.3 Cell-based assay for MOG antibodies

All eight patients underwent testing for MOG antibodies in serum and CSF, and the results are presented in **Table 3**. Three patients showed identical MOG antibody titers in serum and CSF. Three patients had higher serum MOG antibody titers compared with CSF titers, while another two patients exhibited lower serum antibody titers than CSF titers. Notably, Patient 4 displayed negative serum MOG antibody at initial testing, accompanied by a CSF titer of 1:100. This patient experienced clinical relapse six months after self-discontinuing methylprednisolone therapy. Follow-up antibody re-testing revealed a reversed titer profile: the serum MOG antibody turned positive at a titer of 1:10, and the CSF antibody titer declined to 1:1.

**Table 3:** Antibody titers in serum and CSF of 8 patients.

MOG-ab titer	Patient 1	Patient 2	Patient 3	Patient 4	Patient 5	Patient 6	Patient 7	Patient 8
Serum	1:100	1:10	1:32	negative	1:32	1:100	1:100	1:100
CSF	1:100	1:10	1:1	1:100	1:10	1:100	1:320	1:10

#### 3.2.4 Comparative analysis of cytokines in CSF and serum

**Figure 1:**
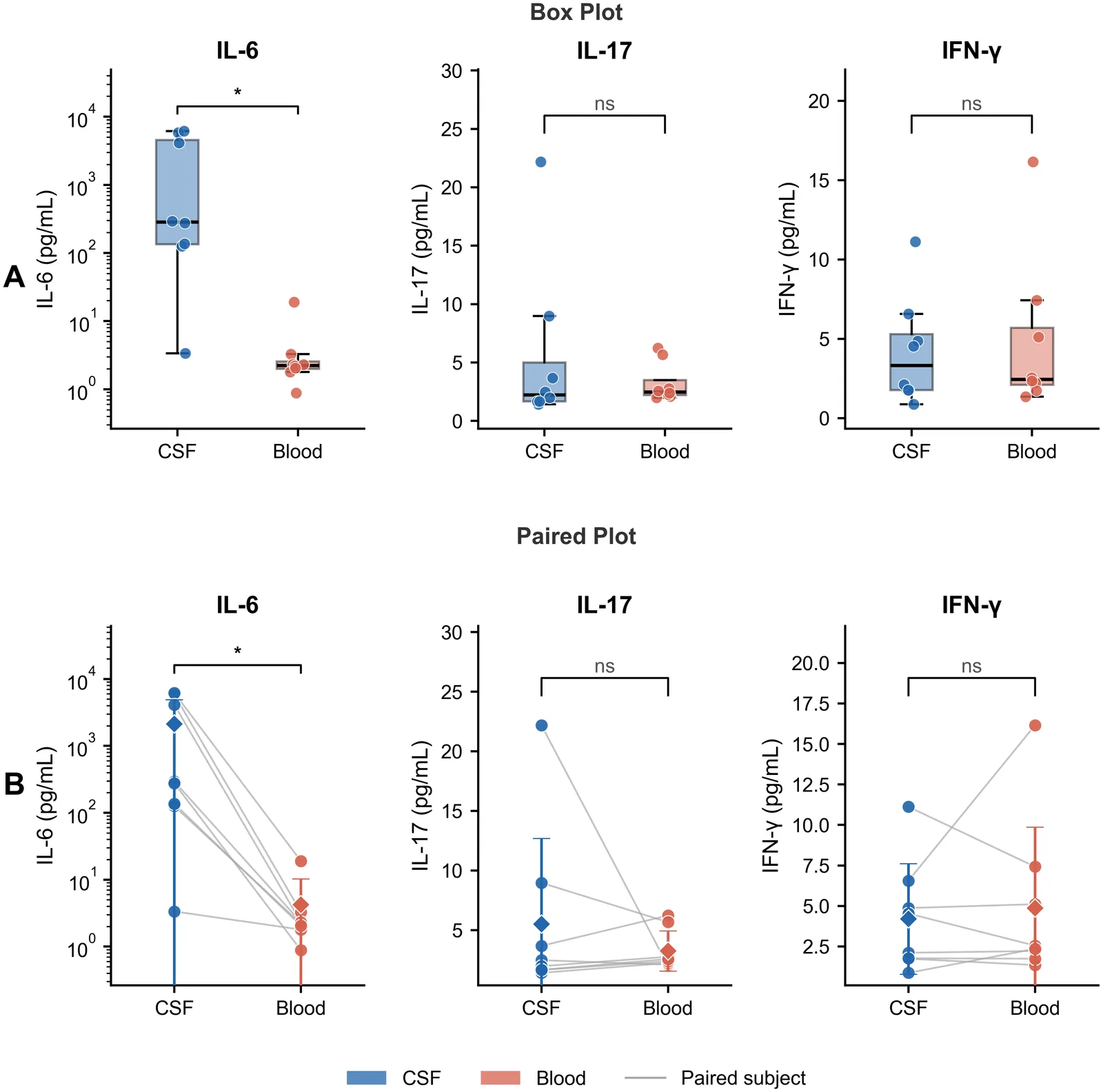
Paired cytokine profiling in CSF and serum from patients with MOG-CCE: **(A)** boxplots show the distribution of IL-6, IL-17, and IFN-γ concentrations in paired CSF and peripheral blood samples. **(B)** Paired scatter plots illustrate individual subject data, with lines connecting CSF and matched blood values. * for p < 0.05, ns for non-significant (P > 0.05).

Cytokine profiling demonstrated that the CSF IL-6 level (median: 284.53 pg/mL; IQR: 3.35–6176.97 pg/mL) was significantly higher than the paired serum level (median: 2.25 pg/mL; Z = 2.521, p = 0.012; Cohen’s d = 1.084). Seven patients (87.5%) had markedly elevated CSF IL-6, including 3 with extremely high levels (>4000 pg/mL). The median CSF-to-serum IL-6 ratio was 220.33 (IQR: 64.26–690.89), and all 8 patients (100%) had a ratio exceeding 1, indicating that CSF IL-6 concentration exceeded the paired serum concentration. By contrast, neither IL-17 (CSF median: 2.23 pg/mL vs. serum: 2.49 pg/mL; Z = 0.280, p = 0.779, Cohen’s d = 0.433) nor IFN-γ (CSF median: 3.32 pg/mL vs. serum: 2.45 pg/mL; Z = 0, p = 1.000, Cohen’s d = 0.156) showed a significant difference between the two compartments (**Figure 1**; **Table 4**).

**Table 4:** Comparative analysis of cytokines in CSF and serum.

Cytokine (N = 8)	CSF median (IQR) (pg/mL)	Blood median (IQR) (pg/mL)	Z-value	P value	Cohen's d
IL-6	284.53 [133.75, 4554.75]	2.25 [1.99, 2.55]	2.521	0.012*	1.084
IL-17	2.23 [1.67, 5.00]	2.49 [2.21, 3.50]	0.280	0.779	0.433
IFN-γ	3.32 [1.76, 5.29]	2.45 [2.10, 5.69]	0.000	1.000	0.156

* for p < 0.05.

### 3.3 Neuroimaging morphological characteristics

#### 3.3.1 MRI intracranial lesion patterns

The intracranial lesion distribution on brain MRI of the 8 MOG-CCE patients is shown in **Table 5**. Cortical lesions were detected in all 8 patients (100%). Leptomeningeal involvement was observed in 7 patients (87.5%), while white matter lesions were present in only 3 cases (37.5%). In terms of lobar lesion distribution, parietal and temporal lobe lesions exhibited equal prevalence, each affecting 5 patients (62.5%). Frontal lobe lesions were identified in 4 patients (50.0%), occipital lobe lesions and bifrontal medial cortex (BFCCE) lesions were both found in 2 patients (25.0%), and brainstem or thalamic lesions were seen in merely 1 patient (12.5%) (**Table 5**).

**Table 5:** MRI intracranial lesion patterns of MOG-CCE patients.

Distribution of intracranial lesion, n (%)	Findings (N = 8)
Cortical involvement	8 (100)
Leptomeningeal involvement	7 (87.5)
White matter involvement	3 (37.5)
Parietal lobe	5 (62.5)
Temporal lobe	5 (62.5)
Frontal lobe	4 (50.0)
Occipital lobe	2 (25.0)
Bifrontal medial cortex (BFCCE)	2 (25.0)
Brainstem/Thalamus	1 (12.5)

#### 3.3.2 Inter-rater reliability of MRI score

The raw scores assigned by radiologist 1 ranged from 2 to 7, with a mean score of 4.25 ± 1.91 and a median score of 4.0 (interquartile range [IQR], 2.8–6.0). The scores provided by radiologist 2 covered the identical range of 2 to 7, with a mean of 4.12 ± 1.96 and a median of 3.5 (IQR, 2.8–6.0). The median value derived from the paired scores of the two raters for each patient was recorded as the consensus median score, which yielded an overall mean of 4.19 ± 1.93, a median of 3.75 (IQR, 2.0–6.0), and a full range of 2 to 7. Complete score concordance between the two radiologists was observed in 7 out of 8 cases; only patient P08 exhibited a minor discrepancy, with scores of 4 and 3 from radiologist 1 and radiologist 2, respectively. A Wilcoxon signed-rank test was performed to compare the paired scoring results, which demonstrated no statistically significant difference between the two raters (Z = 0.000, P = 0.893) (**Table 6**).

**Table 6:** Inter-rater reliability assessment of MRI Scoring System.

Patient ID	Score of radiologist 1	Score of radiologist 2	Median score
P01	6	6	6.0
P02	4	4	4.0
P03	2	2	2.0
P04	3	3	3.0
P05	2	2	2.0
P06	6	6	6.0
P07	7	7	7.0
P08	4	3	3.5
Mean ± SD	4.25 ± 1.91	4.12 ± 1.96	4.19 ± 1.93
Median (IQR)	4.0 (2.8-6.0)	3.5 (2.8-6.0)	3.75(2.0-6.0)
Range	2-7	2-7	2–7

Wilcoxon signed-rank test: Z=0.000, P = 0.893.

#### 3.3.3 Correlation between MRI score and CSF/serum IL-6 ratio

**Figure 2:**
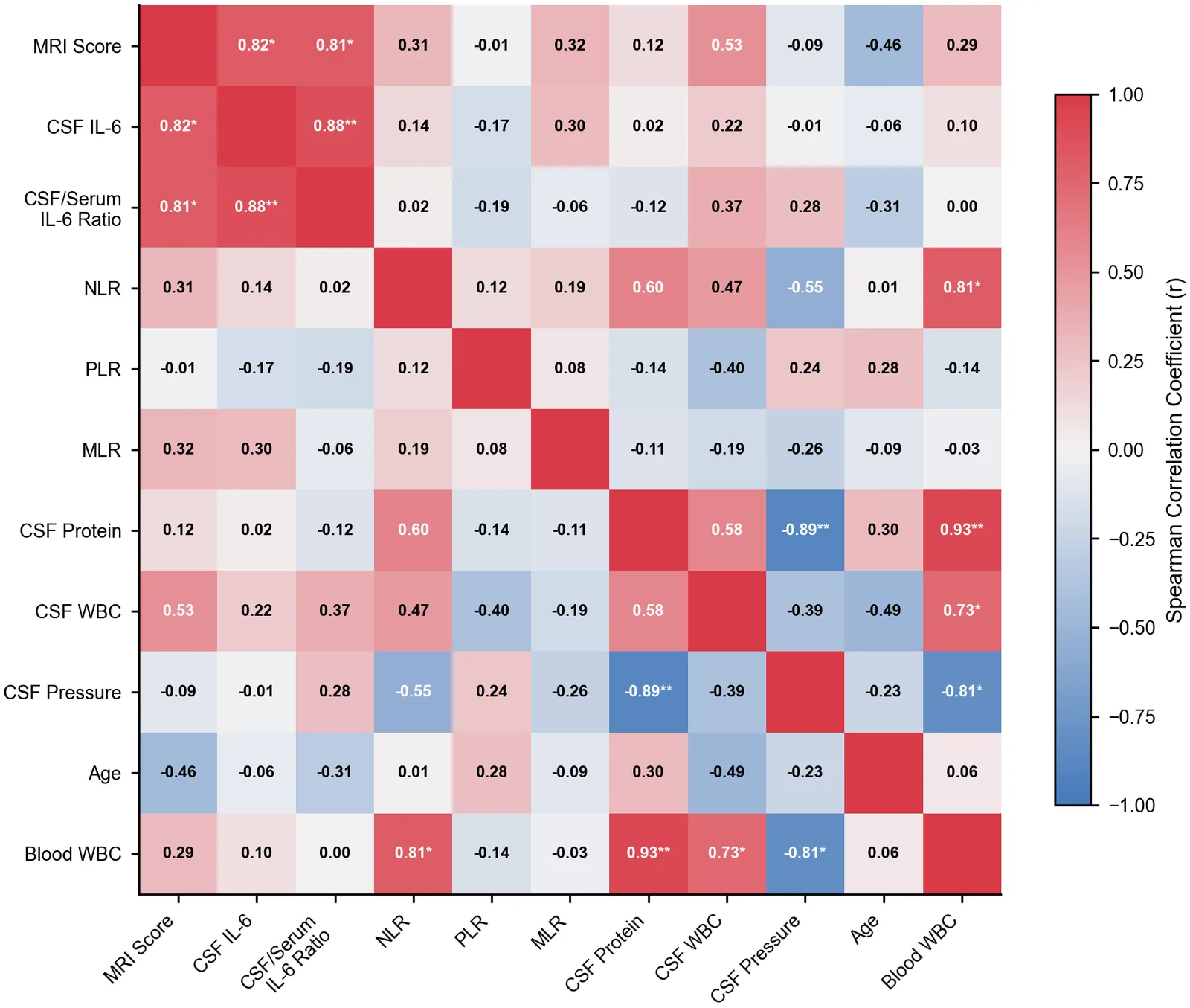
Heatmap of Spearman's rank correlation between MRI score and laboratory parameters in patients with MOG-CCE: This heatmap shows Spearman correlation coefficients linking MRI score to laboratory parameters in MOG-CCE patients. Cell color reflects correlation direction and intensity (red = positive correlation; blue = negative correlation). Values inside cells are correlation coefficient r, with * for p < 0.05 and ** for p < 0.01 statistical significance.

Heatmap of Spearman’s rank correlation revealed that the CSF/serum IL-6 ratio showed a significant positive correlation with MRI score (r = 0.807, p = 0.015), and CSF IL-6 levels also demonstrated a significant positive correlation with MRI score (r = 0.819, p = 0.013). Notably, the CSF/serum IL-6 ratio showed no significant correlation with any conventional systemic inflammatory marker (NLR, PLR, MLR, CSF protein, or CSF leukocyte count), with Spearman rho values ranging from −0.190 to 0.374 (all p > 0.05) (**Figure 2**).

#### 3.3.4 Regression analysis

**Figure 3:**
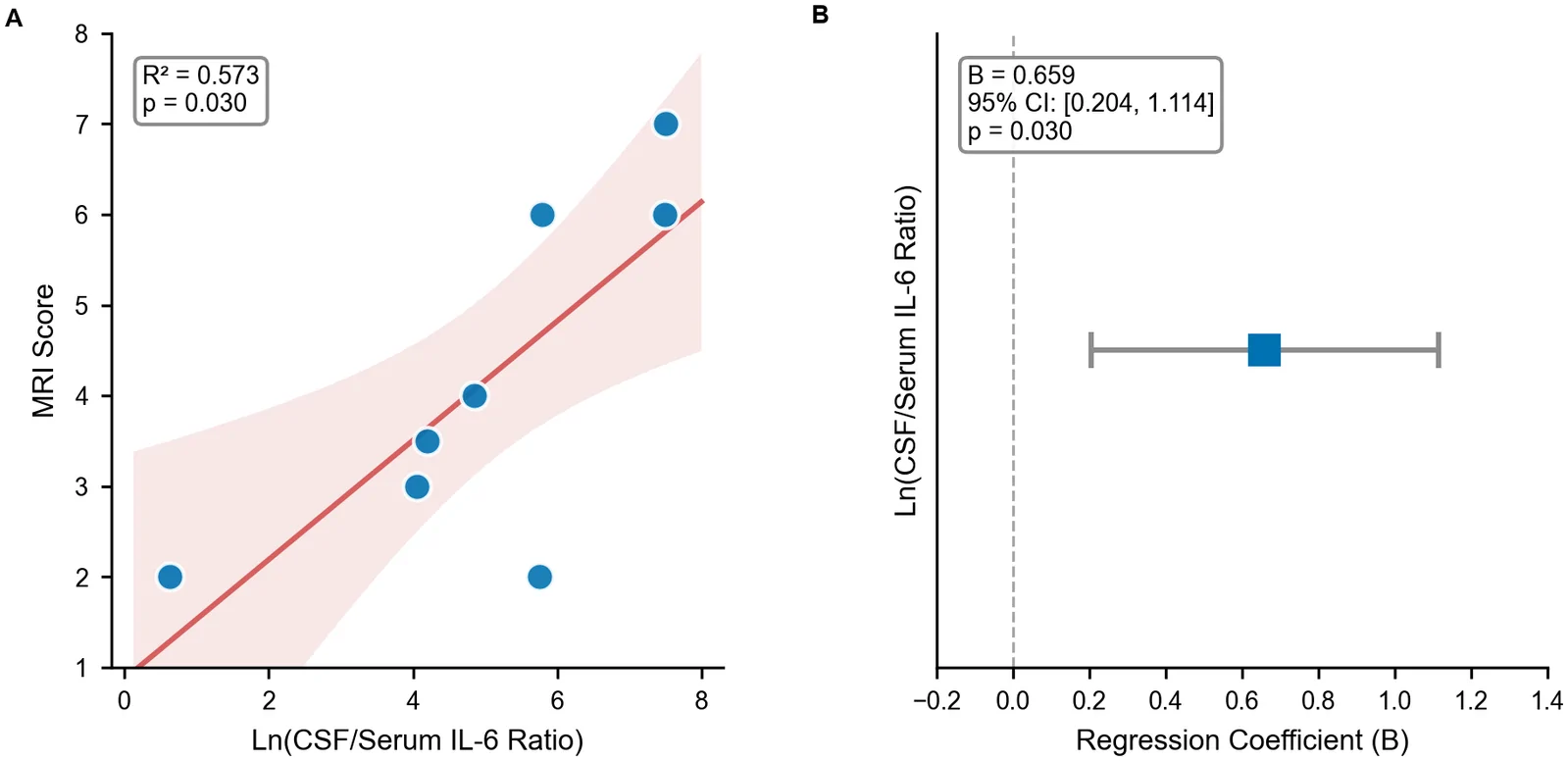
Linear regression analysis of Ln(CSF/Serum IL-6 Ratio) predicting MRI severity scores in MOG-CCE. **(A)** Scatter plot with regression line. The relationship between the natural logarithm of CSF to serum interleukin-6 ratio [Ln(CSF/Serum IL-6)] and MRI semi-quantitative severity scores is shown. The red solid line represents the linear regression fit, and the shaded area indicates the 95% confidence interval. **(B)** Forest plot of regression coefficient. The regression coefficient **(B)** for the association between Ln(CSF/Serum IL-6 ratio) and MRI score is displayed with point estimate (square) and 95% confidence interval (horizontal line).

Simple linear regression demonstrated that the natural-logarithm (Ln)-transformed CSF/serum IL-6 ratio significantly predicted the MRI score (R² = 0.573, adjusted R² = 0.502, F(1,6) = 8.044, p = 0.030), yielding the equation: MRI score = 0.874 + 0.659 × Ln(CSF/serum IL-6 ratio). Each one-log-unit increase in the ratio corresponded to an average increase of 0.659 points in the MRI score (B = 0.659, SE = 0.232, t = 2.836, p = 0.030, β = 0.757). The intercept was 0.874 (SE = 1.263, 95% CI: −2.22 to 4.22; t = 0.692, p = 0.515) and the slope was 0.659 (SE = 0.232, 95% CI: 0.076 to 1.217). Residual normality (Shapiro-Wilk W = 0.876, p = 0.174) and serial correlation (Durbin-Watson = 2.193) tests were non-significant, supporting the adequacy of the model assumptions. Cook’s D values ranged from 0.001 to 1.01; using the 4/n threshold (0.5), one observation approached the cutoff, but residual and leverage diagnostics did not identify it as a strong influential point. Overall, this model accounted for approximately 57.3% of the variance in MRI score. These results should be interpreted as exploratory and hypothesis-generating, requiring validation in larger cohorts (**Figure 3**; **Table 7**).

**Table 7:** Simple Linear regression analysis of the association between Ln(CSF/Serum IL-6 ratio) and MRI score.

Variable	B	SE	95% CI	t	p	β
Intercept	0.874	1.263	[-2.22, 4.22]	0.692	0.515	–
Ln(CSF/Serum IL-6 ratio)	0.659	0.232	[0.076,1.217]	2.836	0.030*	0.757

* for p < 0.05. Residual diagnostics: Shapiro-Wilk W = 0.876, p = 0.174; Durbin-Watson = 2.193; Cook's D range: 0.001–1.01 (no influential points).

Model: R² = 0.573, Adjusted R² = 0.502, F(1,6) = 8.044, p = 0.030.

### 3.4 Treatment and relapse of patients

**Figure 4:**
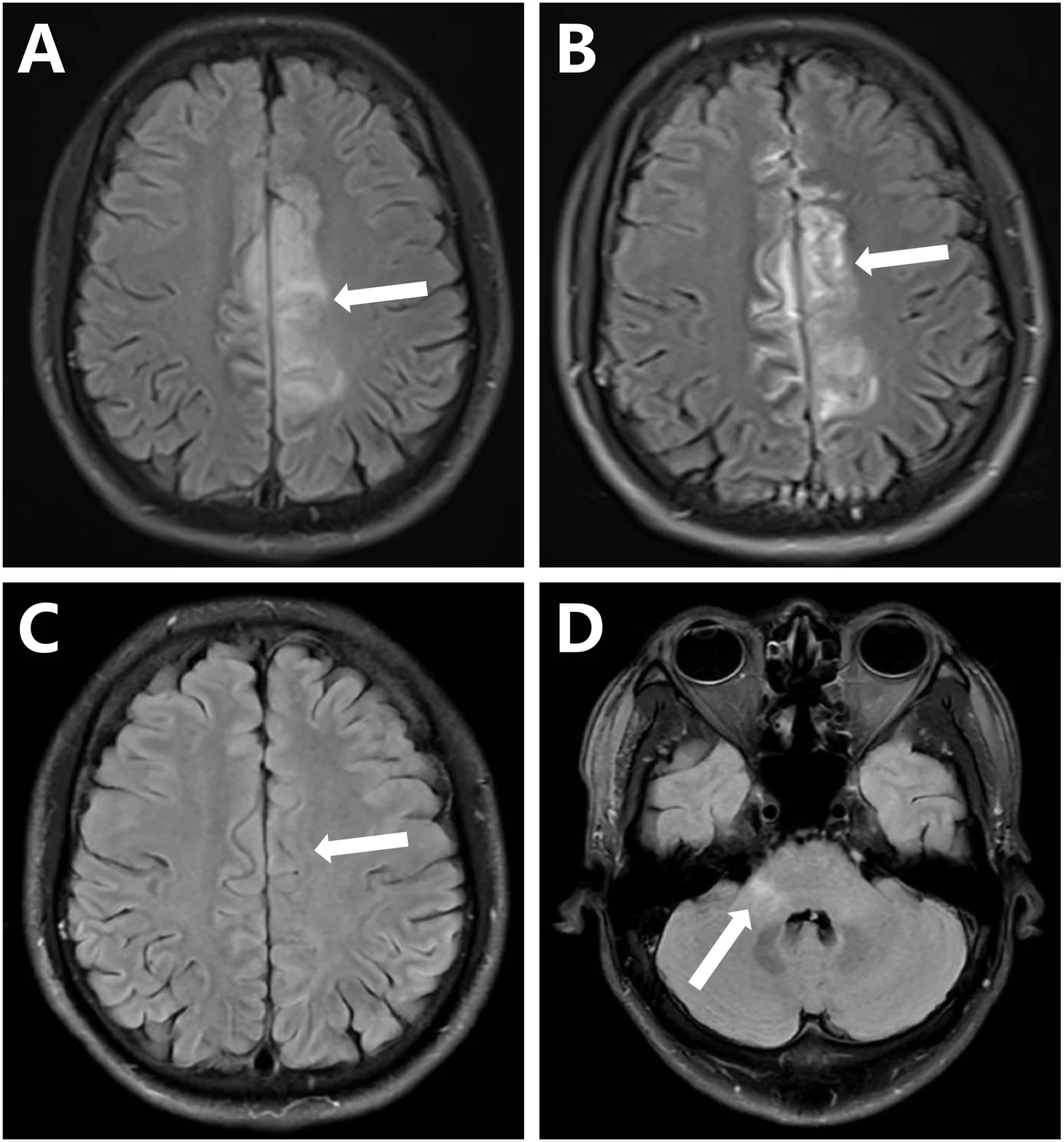
Brain MRI findings at initial onset and relapse in Case 1. **(A, B)** Brain MRI obtained at initial onset. **(A)** T2-FLAIR demonstrates hyperintense signal within the bilateral medial frontal cortex (white arrow). **(B)** Contrast-enhanced T2-FLAIR shows leptomeningeal enhancement over the bilateral medial frontal cortex (white arrow). **(C)** Follow-up T2-FLAIR at relapse demonstrates complete resolution of the previous bilateral medial frontal cortical lesions (white arrow). **(D)** New T2-FLAIR hyperintense lesion is observed in the right middle cerebellar peduncle at relapse (white arrow).

All patients underwent high-dose intravenous methylprednisolone pulse (IVMP) therapy in the acute phase of disease. None received additional plasma exchange (PE) or intravenous immunoglobulin (IVIG). Favorable clinical improvement was observed in all patients after IVMP therapy. Following discharge, all received sequential oral prednisone (60 mg/day) tapered at a rate of 5 mg every two weeks. Four patients were additionally treated with mycophenolate mofetil (MMF, 750 mg twice daily), and one with rituximab (RTX), for relapse prevention. Over a mean follow-up of 9 months (range: 4–48 months), 3 patients (37.5%) experienced relapse. All relapses occurred during corticosteroid tapering or after discontinuation; after adjustment to low-dose maintenance corticosteroid combined with MMF maintenance, no further relapses were observed in these patients through the end of follow-up (**Table 8**). The clinical course of a relapsed case was described as follows: a 34-year-old male had bilateral frontal cortical encephalitis on initial MRI, with symptom remission after IVMP therapy. He voluntarily stopped oral methylprednisolone post-discharge and relapsed 3 months later. Repeat MRI showed a new right pontine brachium lesion with full resolution of frontal lesions. He received a second IVMP therapy followed by maintenance methylprednisolone combined with MMF, with no further relapse (**Figure 4**).

**Table 8:** Treatment and Relapse of patients.

Treatment and Relapse	Findings (N = 8)
Acute-phase IVMP therapy, n (%)	8 (100)
Maintenance therapy, n (%)	8 (100)
Methylprednisolone monotherapy, n (%)	3 (37.5)
Methylprednisolone + MMF combination therapy, n (%)	4 (50)
Methylprednisolone + RTX combination therapy, n (%)	1 (12.5)
Relapse during follow-up, n (%)	3 (37.5)
Median follow-up duration, months (range)	9 (4 – 48)

## 4 Discussion

In this exploratory, hypothesis-generating study, we systematically characterized the clinical features, CSF cytokine profiles, and MRI findings of 8 adolescent and adult patients with MOG-CCE. The central finding was a significant positive correlation between CSF IL-6 levels and semi-quantitative MRI score (r = 0.819, p = 0.013), with regression analysis further confirming that the CSF/serum IL-6 ratio predicted the extent of MRI lesions (R² = 0.573). Together, these data provide quantitative evidence supporting IL-6 as a candidate biomarker of inflammatory severity in MOG-CCE. From a pathophysiological standpoint, the principal contribution of this study is the demonstration—in a well-characterized MOG-CCE cohort and consistent with other CNS immune-mediated disorders—that IL-6 is locally produced and compartmentalized within the CNS, independent of peripheral production (all patients had a CSF/serum IL-6 ratio > 1, and the ratio was uncorrelated with systemic inflammatory markers), and that the degree of this intrathecal enrichment quantitatively correlates with the extent of radiological involvement.

Clinically, all 8 patients (100%) presented with seizures at onset, aligning with the findings of Cao et al. (11) and Tang et al. (12). In their multicenter study, Cao et al. identified seizure as the core clinical hallmark of the cortical encephalitis phenotype in adult MOGAD, distinguishing it from the predominantly ADEM-like presentation of pediatric MOGAD (11). Our data reaffirm and extend this clinical spectrum: male patients accounted for 75% of our cohort, consistent with the male predominance reported by Wu et al. (13). Supporting the concept of meningeal–cortical involvement proposed by Budhram et al. (3), 75% of our patients had positive meningeal signs, and 87.5% showed leptomeningeal enhancement on MRI—indicating that MOG-CCE involves not only the cerebral cortex but also the meninges.

Turning to the mechanistic underpinnings of the core association, the most clinically significant observation was the robust positive correlation between CSF IL-6 and MRI score (r = 0.819, p = 0.013) and the predictive value of the ratio confirmed by regression (R² = 0.573). Notably, patients with extremely high CSF IL-6 (>4000 pg/mL) tended to have lesions extending into deep white matter and/or the brainstem—a pattern that merits validation in larger cohorts. Critically, this association must be interpreted mechanistically rather than as a mere statistical observation. CSF IL-6 levels (median 284.53 pg/mL) exceeded paired serum levels (2.25 pg/mL) by more than two orders of magnitude, with a Cohen’s d of 1.084, arguing against a purely systemic origin. Whether this enrichment reflects bona fide intrathecal synthesis, however, remains to be determined by Reiber graph analysis (8); although the ratio > 1 and its dissociation from systemic markers support compartmentalized CNS activation, intrathecal synthesis remains unconfirmed. Linking this unresolved question to known biology, IL-6 activates the JAK/STAT3 pathway upon receptor binding, promoting B-cell differentiation and Th17 polarization and thereby establishing a positive-feedback cytokine amplification loop within the CNS—a mechanism that echoes the autoimmune encephalitis findings of Levraut et al. (7). What distinguishes the present study is that we went beyond documenting CSF IL-6 enrichment: using regression analysis, we established a quantitative predictive link between this enrichment and the extent of imaging injury, moving IL-6 from an accompanying marker of inflammatory activity to a candidate biomarker of lesional burden. The cross-sectional design precludes causal inference, and we acknowledge this limitation. Of note, recent work has further substantiated CSF IL-6 as a CNS inflammation marker across disease spectra: in progressive multiple sclerosis, for instance, CSF IL-6 levels correlate positively with EDSS scores (14).

Synthesizing these observations, the CSF/serum IL-6 ratio was uncorrelated with conventional systemic inflammatory markers (NLR, PLR, MLR, CSF protein, and CSF leukocyte count) yet was significantly positively correlated with MRI score. This dissociation supports the interpretation that the ratio reflects CNS-compartmentalized immune activation rather than systemic inflammation, strengthening its candidacy as a disease-specific biomarker for MOG-CCE. The value of CSF IL-6 as an indicator of CNS-compartmentalized inflammation has been recognized in neuroimmune diseases more broadly (15); our data extend this concept to MOG-CCE, providing quantitative evidence for IL-6 as a candidate biomarker within the evolving MOGAD biomarker landscape.

Convergent evidence comes from the subset of patients with extremely high CSF IL-6 (>4000 pg/mL), whose MRI lesions extended beyond the cortex into white matter and/or brainstem—suggesting that extreme IL-6 elevation may herald widespread inflammatory injury. Andersen et al. (16) proposed that sustained local pro-inflammatory cytokine activation within the CNS drives inflammation from the cortex to deep structures; our imaging and biomarker data provide quantitative support for this model. Independently, Wang et al. (17) demonstrated the biomarker value of CSF HMGB1 in pediatric MOGAD, and our findings complement this by supporting CSF IL-6 as a candidate quantitative biomarker in the adolescent/adult population.

Turning to the methodological aspects, our MRI scoring system provides a semi-quantitative tool for grading the extent of involvement in MOG-CCE. The 87.5% concordance rate and non-significant Wilcoxon test (P = 0.893) indicate good reproducibility between two independent raters—a prerequisite for any imaging-based biological association analysis, as the validity of such analyses rests on the stability and consistency of the scoring instrument (10). The parietal and temporal lobes were the most commonly affected (62.5% each), consistent with Pace et al. (18); FLAIR was the most sensitive sequence for detecting cortical lesions, in line with Liu et al. (19) and Budhram et al. (3).

From a clinical perspective, the CSF/serum IL-6 ratio and MRI score are exploratory candidates for risk stratification that could help identify patients who may benefit from earlier maintenance immunotherapy. However, several confounders must be acknowledged: BBB permeability was not measured (QAlb/Reiber unavailable), sampling occurred only in the acute phase, and concomitant corticosteroid exposure may modulate cytokine levels. Notably, unlike IL-6, neither IL-17 nor IFN-γ showed a CSF–serum gradient (both p > 0.7), suggesting these cytokines are not compartmentalized within the CNS in MOG-CCE; the limited sample precludes firm conclusions about smaller effects. Regarding relapse prediction, an exploratory Mann–Whitney U comparison of relapsers (n = 3, median Ln ratio = 4.20, median MRI score = 3.5) versus non-relapsers (n = 5, median Ln ratio = 5.75, median MRI score = 4.0) yielded no significant differences for either Ln ratio (U = 6.0, exact p = 0.393, rank-biserial r = 0.20) or MRI score (U = 7.5, p = 1.000, rank-biserial r = 0.00). The small and null effect sizes indicate that, at the current sample size, the CSF/serum IL-6 ratio and MRI score do not reliably distinguish relapsers from non-relapsers. All three relapses occurred during or after rapid corticosteroid taper, suggesting that treatment intensity—rather than baseline biomarker levels—may be the primary driver of early relapse in this cohort. Of note, both patients with the BFCCE subtype relapsed (2/2, 100%), compared with 1/6 non-BFCCE patients, generating a descriptive signal that the BFCCE phenotype may constitute a risk-enriching substrate independent of absolute IL-6 levels; this observation requires prospective validation in larger cohorts.

Regarding the BFCCE subtype, both patients with this subtype relapsed, compared with only 1 of 6 non-BFCCE patients. Although the numerical difference (100% vs. 17%) carries clinical signal, the sample (n = 2) precludes a statistical conclusion that BFCCE is an independent risk factor for relapse. The combination of the BFCCE phenotype and high IL-6 levels may constitute a hypothesis-generating risk signature warranting prospective evaluation. The original description by Fujimori et al. (20) provides directional support from a larger sample, and the clinical vulnerability of BFCCE patients during the corticosteroid tapering window warrants prospective evaluation.

In terms of treatment and outcomes, all patients responded acutely to high-dose intravenous methylprednisolone, consistent with the efficacy data from Trewin et al.’s global review of 4699 MOGAD patients (21). Nevertheless, the 37.5% relapse rate during follow-up is non-trivial. Notably, all relapses occurred in patients on corticosteroid monotherapy, whereas none occurred in those receiving mycophenolate mofetil or rituximab—a pattern that aligns with recent international guidelines advocating long-term maintenance immunotherapy in MOGAD (21, 22). An intriguing observation is that none of the relapses recurred as the cortical encephalitis phenotype; instead, they manifested as new brainstem or white matter lesions, suggesting that MOGAD relapses may exhibit phenotype switching across episodes. The systematic review by Andersen et al. (23) underscored the importance of early identification of patients at high risk for relapse; our data suggest that the combination of the BFCCE subtype and high IL-6 levels may constitute a hypothesis-generating risk signature requiring prospective validation.

These findings must be interpreted within several limitations. First, the single-center retrospective design with a small sample (n = 8) limited statistical power; the regression model had only 6 degrees of freedom, and the marginal p value (0.030) carries a risk of sample-size-dependent false positivity. Furthermore, 42.7% of MRI score variance remains unexplained, suggesting that additional predictors (e.g., MOG-IgG titer, disease duration) merit inclusion in future multivariate models. Second, although the MRI scoring system showed acceptable inter-rater reliability (87.5% concordance; P = 0.893), more rigorous metrics (ICC, weighted Kappa) could not be calculated given n < 15 (10). Third, the absence of blood-brain barrier permeability indices (e.g., QAlb) precluded definitive Reiber graph analysis (8); although the CSF/serum IL-6 ratio > 1 and its dissociation from systemic markers suggest CNS-compartmentalized activation, intrathecal synthesis remains unconfirmed. Fourth, the cross-sectional design precludes causal inference.

Future research should prioritize: (1) multicenter prospective cohort studies to validate the CSF/serum IL-6 ratio as a severity biomarker in larger MOG-CCE populations; (2) Reiber graph analysis to determine whether CSF IL-6 elevation reflects intrathecal synthesis or blood-brain barrier disruption; (3) prospective validation of the MRI scoring system with calculation of ICC or weighted Kappa; (4) integration of IL-6 levels into a risk stratification framework to guide individualized treatment decisions.

## 5 Conclusion

In this exploratory, hypothesis-generating pilot study of eight adolescent and adult patients with MOG antibody-associated cerebral cortical encephalitis (MOG-CCE), CSF IL-6 levels correlated positively with semi-quantitative MRI severity scores, and the CSF/serum IL-6 ratio accounted for approximately 57.3% of the variance in MRI score. The ratio was not correlated with conventional systemic inflammatory markers, suggesting—rather than proving—compartmentalized elevation of IL-6 within the CNS; formal confirmation of intrathecal synthesis requires Reiber graph analysis, which could not be performed in this retrospective cohort. At the current sample size, neither the ratio nor the MRI score reliably distinguished relapsing from non-relapsing patients, and all relapses occurred during corticosteroid tapering, underscoring the importance of maintenance immunotherapy. These findings support the CSF/serum IL-6 ratio as a candidate severity biomarker that warrants prospective validation in larger multicenter cohorts with standardized clinical severity assessment and Reiber analysis.

## Data Availability

The original contributions presented in the study are included in the article/supplementary material. Further inquiries can be directed to the corresponding author.
